# Comparison in the efficiency of different murine lines for genotoxicity assays

**DOI:** 10.2478/v10102-012-0023-4

**Published:** 2012-09

**Authors:** Daniel Francisco Arencibia-Arrebola, Luis Alfredo Rosario-Fernández, Yolanda Emilia Suárez-Fernández, Alexis Vidal-Novoa, Livan Delgado-Roche

**Affiliations:** 1 Finlay Institute, Havana, Cuba; 2 Institute of Pharmacy and Food Science, Havana, Cuba; 3 Agrarian University of Havana, Veterinary Faculty, Havana, Cuba; 4 University of Havana, Biology Faculty, Havana, Cuba; 5 Center of Study for the Research and Biological Evaluations (CEIEB-U.H), Havana, Cuba

**Keywords:** efficiency, murine lines, rats, mice, genotoxicity, genetic toxicology

## Abstract

The aim of this research was to compare the efficiency of different murine lines for genotoxicity assays. Rats and mice of different murine lines were used. The spontaneous and induced indexes were evaluated according to alkaline comet assay of peripheral blood leukocytes, micronucleus and chromosomic aberration assay of bone marrow cell, and sperm head morphology assay. In most of the evaluated assays the line of Balb/c mice turned out to be the ideal biomodel, with less spontaneous indexes and high induced indexes to the mutagen used; allowing to detect in a narrow error margin those substances that are classified of very low genotoxicity. These results demonstrate that genetically the line of Balb/c mice in both sexes is more stable than the other ones evaluated. This suggests the use of the Balb/c line on in vivo genotoxicity assay will increase sensibility and robustness.

## Introduction

It is concluded that the mutagenic and genotoxic studies in experimental toxicology have been completed when all the studies of toxicity including sharp potential of the product to evaluate in two species and for at least two pathways of administration in an independent way have been undertaken (Arencibia *et al.*, 2009). The studies done show risk associated to direct or indirect damage on the genetic material, where a positive response is associated to the consideration of the product for further evaluation. A wide range of *in vivo* and *in vitro* assays have been described to detect genotoxicity at different expression stages, all with a high sensibility and specificity (Loeb & Loeb, 2000).

The main problem is that the researchers use the different mouse and rat genetic lines of the available biomodel for convenience. Nevertheless genetic differences among the lines of the biomodel, present differentiation in the expression due to genome damage (Arencibia *et al.*, 2011). In most instances however this decision does not have a theoretical-practical basis that would be in line with the selection. This is conditioned by lack of studies in this subject and could lead to inability to compare results obtained by different research groups on similar products or of the same group, in different moments.

The classic mutagen more used on *in vivo* genotoxicity studies are the cyclophosphamide (CF), administered by intraperitoneal route (i.p). The CF is an alquilant agent that forms monoadducts and crossed connections between chains as consequence of the appearance of ruptures for reparative mechanisms effects. This drug is used with great effectiveness as an antineoplastic (Arencibia *et al.*, 2011a). The CF is a cloroethylamine, considered a bifunctional alquilant without specificity for some of the cell cycle phase (Arencibia *et al.*, 2011a).

The present approach to this kind of work classifies appropriately only very safe or very genotoxic products, but those provoking less damage are improperly classified. Indeed it is important to perform a comparative study of the spontaneous and induced rates of genotoxicity in the genetic lines of mice and rats available and most used in research worldwide at the different expression levels with different cytogenetic methods. This will contribute to provide genetic toxicologists with a statistically validated method, when selecting the genetic line of mice and rats to perform the mutagenesis and carcinogenesis studies.

The assessment of this research were:

1. Assess the efficiency in the use of the lines of Balb/c, NMRI, OF-1 and C57/BL6/Cenp mice of both sexes in the alkaline comet assay of peripheral blood leukocytes, micronucleus and chromosomic aberrations in cells of the bone marrow and the sperm head morphology assay.
2. Assess the efficiency in the use of the lines of rats Sprague Dawley (SD), Lewis and Wistar of both sexes in the alkaline comet assay of peripheral blood leukocytes, micronucleus and chromosomic aberrations in cells of the bone marrow and the sperm head morphology assay.
3. To compare the mouse line and rat selected of both sexes in the alkaline comet assay of peripheral blood leukocytes, micronucleus and chromosomic aberrations in cells of the bone marrow and the sperm head morphology assay.

## Materials and methods

### Animals and Experimental Conditions

Lines of mice were Balb/c, NMRI, OF-1 and C57/BL6/Cenp of both sexes, 8–9 weeks of age, body weight 26–30 g at the end of the quarantine. Rats of the genetic lines SD, Lewis and Wistar were used, 6–8 weeks, 180–210 g at the end of the quarantine. Animals were randomly distributed in groups of 5 animals each, with 10 animals in total (in the two replics).

### Experimental Groups

The experimental groups were given: group 1 nothing (negative control), group 2: Tween 65 at 2%, group 3: NaCl to 0.9%, group 4 (positive control): CF in dose of 50 mg/kg, via intraperitoneal (i.p), (Ledoxina^®^, Lemery, CORP), which was diluted in saline solution (NaCl) to 0.9%. The solution was administered immediately after being prepared, 24 than 48 hours and then at the 24 hours before the euthanasia at 10 ml/kg (Shayne, 2007). Substances were administered via oral at 2 ml/kg during a period of 14 days, prepared 2 hours before the administration (Shayne, 2007). All groups of animals were exposed to the same handling conditions by the technique of gastric intubation during a period of 14 days.

We must highlight that for the case of the sperm head morphology assay we used mice and male rats of each evaluated line, forming these same experimental groups to the dose and mentioned previously, the administration of each one of these substances was during 5 consecutive days and then received no treatment 35 days (mice) (Shayne, 2007) and 52 days (rats) (duration of the spermatic cycle of each species) (Shayne, 2007).

At the end of experiment we were able to do the alkaline electrophoresis of individual cells assay (comet assay), micronucleus assay in bone marrow, chromosomic aberrations assay in bone marrow and the sperm head morphology assay as previously reported (Arencibia *et al.*, 2009a; Arencibia & Rosario, 2010).

### Cytogenetics

#### Alkaline electrophoresis of individual cells

The comet assay of peripheral blood leukocytes was performed according to (Arencibia & Rosario, 2010). 15–20 µL of samples were suspended in 140 µL of low melting point agarose to 0.5%; then previously prepared sheets were added with agarose. They dove in lysis solution to pH 10 for 1.5 hr to 4 °C and subjected to 20 min of denaturalization in electrophoresis regulatory solution, pH>13. The electrophoresis was performed to 300 mA and 1 V/cm during 18 to 20 min (Collins, 2004). The sheets were washed with solution neutralization regulatory using the Tris 0.4 M to pH 7.5 and clarified with distilled water, later they were tinted with silver nitrate to 0.05%. The nucleoids were evaluated using a microscope of light transmission, Olympus BH-2, by three independent observers, to establish an average among readings. The visual analysis includes the quantification of 100 comets per animal in the gel center. The comets were classified corresponding to the category or degree of corresponding to DNA damage between 0 and 4 category (Collins, 2004; Flee & Steinert, 2003). The magnitude of the DNA damage was expressed in arbitrary units (UA) starting from possible values in an interval of 0–400.

#### The micronucleus assay in bone marrow cells

The micronucleus assay in bone marrow cells were performed according to the standardized protocols and adjusted by (Arencibia *et al.*, 2009a). One medullar femur cavity/animal was washed with 3 mL of fetal bovine serum. The bone marrow cells obtained were centrifuged at 200 g for 10 min; and cell pellet was expanded in coverslips. A minimum of 2 sheets/animal were mounted, kept 24 hr at room temperature for drying and fixing in absolute methanol (5 min). They were tinted in Giemsa at 5% during 12–15 min. The analysis was performed for three independent observers, using a microscope Olympus BH-2 (100X with immersion lens). The presence of polychromatic erythrocytes (PE) and normochromatic erythrocytes (NE) in 2000 cells/animal were counted (Hayashi *et al.*, 1994). Also, the frequency of PE with micronucleus (MN-EP) was calculated in 2000 PE/animal. Later on the cytotoxicity index was calculated (PE/NE) of the total population of erythrocytes and the number of PE were counted with 1 MN, 2 MN and >2 MN per treatments group (Hayashi *et al.*, 1994).

#### Chromosomic aberration assay in bone marrow cells

The chromosomic aberration assay in bone marrow cells were performed according to the standardized protocols and adjusted by (Arencibia *et al.*, 2009a). The cellular division in metaphase stopped using colchicine (4 mg/kg, via i.p), in the next day schedule (4 hours before the euthanasia). One medullar femur cavity/animal was extracted and washed with 3 mL of foetal bovine serum (FBS). The cellular suspension was centrifuged, hypotonic solution (KCL, 0.075 M), was added to pelleted cells. After centrifugation pelleted cells were fixed in mixture methanol-glacial acetic acid (3:1 proportion), 15 minutes (3×) and next extended in humid sheets with previous cooling. The sheets dried off to the air and they were tinted with Giemsa 10% solution, 30–35 min and 100 metaphases counted/ animal, number of cells with aberrations (ruptures and chromosomes exchanges, ruptures and chromatids exchanges) and gaps frequency (Kramer, 2000). Also the mitotic index MI% was calculated (metaphases percentage in 1 000 readable cells) and the number of polyploidy cells in 1 000 readable cells. All the determinations were read by two observers, and then an average established (Arencibia *et al.*, 2009a).

### Sperm head morphology assay

In mice both epididymis and in rats one epididymis was extracted, minced into Petri dishes that contained 3 mL of isotonic solution of NaCl 0.9%. The sample was homogenized with Pasteur pipettes (Arencibia *et al.*, 2009a).

#### Count of sperms

0.05 mL trypsin 0.25% was added to homogenates, after five minutes more than 2 mL of NaCl 0.9% was added. A dilution of the homogenized with trypsin in NaCl-formaldehyde to 1% (1:10) was done and counts were done with a NewBauer chamber and microscope Olympus BH-2, 10× (Wyrobek, 1983; Arencibia *et al.*, 2009a).

#### Sperm head morphology

Five eosin drops to 1% were added to the homogenized dilution, and incubated 5 minutes. Later on, a drop was mounted on a dry sheet (Arencibia *et al.*, 2009a). 500 sperms were analysed (minimum: 2/animal) with a microscope Olympus BH-2, at 40×. The observations were performed blindly by two independent observers, and an average was then established. The classification was based on normal and abnormal heads that it includes amorphous, banana, without hook and with two tails (Arencibia *et al.*, 2009a).

#### Analysed variables

The analysed variables were the spermatic concentration in epididymis, spontaneous frequency of anomalous sperms heads, erythrocytes number in bone marrow with micronucleus, cytotoxicity index (relationship among young erythrocytes/mature erythrocytes), total of cells with structural aberrations in the chromosomes, mitotic index (number of cells that were in the phase of cellular division of metaphase) and the per cent of peripheral blood leukocytes, for levels of damage in the DNA according to level 1, 2, 3, 4 of less to more damage (Arencibia *et al.,* 2009a; Arencibia & Rosario, 2010).

### Euthanasia methods and statistical analysis

The method of euthanasia selected was the cervical dislocation with previous ether atmosphere. All the results were compared against the group negative control and among lines of mice and of rats for the same group and sex. Likewise the results were compared among murine species (Arencibia *et al.*, 2009a; Arencibia & Rosario, 2010). The continue variables were analysed by ANOVA test (*p<*0.05) and the categorical variables by Chi Squared (*p<*0.01) (Arencibia *et al.*, 2009a; Arencibia & Rosario, 2010). All the analyses were performed using the Statsoft for Windows. StatSoft, Inc. (2003). STATISTICA (data analysis, software system), version 6.

### Permission of animal ethics committee

During the experimental process the established ethical principles were respected for the research with laboratory animals. The authors declare that this work was made on the base of good practices of preclinical laboratory present in the national regulation of protocols approval of research in the Cuban republic. It is also declared on the part of the authors that it was obtained the consent of protocol approval and report in writing when this research began.

## Results

The comparison among lines of mice hurtled as a result that the Balb/c line in both sexes differed with the other lines evaluated in the four assay performed in this study. Lowest rate in genotoxic damage was obtained with the mutagen used. In Balb/c mice the nucleoids per cent with 0 degree of damage in the DNA according to the comet assay was 49.97–57.47% in both sexes, however the CF induced a considerable decrease being values among 23.33–25.80%. When being evaluated this line in the micronucleus assay, spontaneous indexes of the per cent of polychromatic erythrocytes were obtained, with results between 0.13–0.18%. Equally the CF induced high per cents with values among 1.65–1.82%. The chromosome aberration assay detects endogenous values up to 7–10 total cells with aberrations. The CF induced a total of 175–192 cells with aberrations in bone marrow cells. When evaluating male mice of this line in the sperm head morphology assay, the total of normal heads was obtained with values among 483.2–489.3 in 500 cells registered on the other hand the CF cause a significant decrease with values among 387.2–411.8.

On the other hand in the comparison among lines of rats was obtained that the SD rats in both sexes differed with the other evaluated lines keeping in mind the spontaneous and induced rates of damage in the variables analysed in this study. In the comet assay of peripheral leukocytes it was obtained in this line of rats endogenous values among 78.21–80.46 of the nucleoids per cent with 0 degree of damage to the DNA. However the mutagen used diminished these results significantly with values among 32.76–35.56% in the 0 degree of nucleoids. The evaluation of this line in the micronucleus assay detects endogenous rates among 0.15–0.22% of micronucleus in polychromatic erythrocytes. Inducing the CF rates among 1.68–1.74% of micronucleus in polychromatic erythrocytes in both sexes. It was detected basally among 17–23 total cells with chromosomic aberrations; the CF induced a total of 220–246 cells with chromosomic aberrations in bone marrow in both sexes. When being evaluated this line of rats in the sperm head morphology assay, it showed results among 455.3–460.6 of normal heads in 500 total cells, differing these results with those obtained with the administration of the CF. The CF mutagen induced among 85–99 anomalous heads in 500 registrable cells.

In the comparison among mice and rat species selected, the Balb/c mice were the most efficient in all evaluated assay, except in the comet assay. In this assay the rats demonstrated less endogenous damage and bigger susceptibility to the CF.

## Discussion

When comparing the endogenous and induced results among both murine species, it was obtained as a result that in most of the evaluated assay the line of Balb/c mice turned out to be the ideal biomodel, being the lower spontaneous indexes and induced high to the mutagen used; allowing us to detect in a narrow error margin those substances that are classified of very low genotoxicity. The results obtained in this line of mice differed significantly with those obtained in SD rats in both sexes. Only in the comet assay of peripheral blood leukocytes, was it obtained in rats of the SD line lower endogenous values that in Balb/c mice.

When performed a comparison among lines of mice keeping in mind the results obtained in the alkaline comet assay, it is appreciated that there were significant differences among the Balb/c line in both sexes with the other ones, it didn't seize among the other three evaluated lines. These results were obtained when comparing the % of spontaneous nucleoids and induced with CF in each one of the levels of damage, being in this line the lower spontaneous or basal levels of damage and acceptable induced, standing out their sensibility to mutagenic substances as the CF (Arencibia *et al.*, 2010, 2011a).

From the micronucleus assay it was obtained as a result that the line Balb/c equally differed with the other three lines evaluated in both sexes. The differences were given when keeping in mind the cytotoxicity index obtained of the relationship polychromatic erythrocytes (PE)/normochromatic erythrocytes (NE), the genotoxicity index (% of PE that contain micronucleus), as well as the number of PE with 1, 2 or more than 2 micronucleus, as index of damage severity. In this line we obtained the lower spontaneous results and the highest induced results, being observed a high sensibility of this animal biomodel to detect clastogenic compound (Arencibia *et al.*, 2009d, 2009e, 2010, 2010a, 2011b).

On the other hand, in the chromosomic aberration assay, useful to detect *in vivo* substances that induce aberrations of structural type in bone marrow cells, met significant differences among the Balb/c line in both sexes with the other evaluated lines. When performed the comparison among lines it was obtained that the Balb/c differed from the other ones, for the fact of having the lower spontaneous indexes and induced intermissions keeping in mind the total cells with aberrations, number of chromosomic aberrations and chromatid aberrations, aberrations of the gaps type, as well as the number of cells with polyploidies and the mitotic index. These last two variables are of the numeric type (Arencibia *et al.*, 2009f, 2010b, 2010c, 2010d). The line of OF-1 mice also differed in significant way of the C57BL/6/cenp, in this last one the highest spontaneous and induced values were obtained, being less efficient and sensitive to the damage determined by this cytogenetic technical (Arencibia *et al.*, 2010b).

When performed a comparison among these 4 lines in male mice, in the sperm head morphology assay, it was obtained that the most efficient line again was the Balb/c (Arencibia *et al.*, 2009c, 2009e, 2010). This line differed in an evaluated significant way of the other ones, keeping in mind that in this line the highest values in spontaneous spermatic concentration were obtained as cytotoxicity index and the lowest values in anomalies in the sperm head as indicator of genotoxic damage (Arencibia *et al.*, 2009e, 2010, 2010d; Arencibia, 2010). Of equal forms it was the line that better responded to the evaluated mutagen (Arencibia *et al.*, 2009e, Arencibia & Rosario, 2011). The spontaneous results obtained in Balb/c mice are lower than those obtained by us in the negative control group (not administered), in a study where they were used OF-1 mice (Arencibia *et al.*, 2009g). These results demonstrate that genetically the line of Balb/c mice in both sexes is more stable than the other ones evaluated, besides demonstrating that it presents an acceptable response to the action of the mutagenic substances.

Comparing the lines of rats less damage was observed at the basal DNA in the line of SD rats in both sexes when being evaluated in the alkaline comet assay of individual cells (Arencibia & Rosario, 2010a; Arencibia *et al.*, 2010e, 2011c). The induction results obtained with the CF did not differ among lines, result that was manifest in both sexes.

The CF differed again with the other groups in the three lines of rats evaluated in the micronucleus assay. The lower basal result of cytotoxicity given by the relationship PE/NE was obtained in the line of SD rats in both sexes (Arencibia & Rosario, 2010b, 2010c; Arencibia *et al.*, 2011c, 2011d). Equally the response of this rat line to the CF was high, but less as compared to the clastogenic results obtained in the Lewis and Wistar lines in both sexes. On the other hand the CF induced a considerable number of MN in this line of rats but the biggest clastogenic effect it was in the line of Wistar rats in males.

The results of the chromosomic aberrations assay, demonstrated once again the use of SD rats as more efficient biomodels in the genotoxicity assay. Again the analysed variables keeping in mind the aberrations of structural type and the mitotic index differed among the SD rats in both sexes when being compared with the Lewis and Wistar lines. We obtained the lowest basal results in cells with aberrations in SD rats (Arencibia & Rosario, 2010b, 2010d). But the biggest effect in the CF it was obtained in the line of Lewis rats.

The male SD rats overcame the other evaluated lines in the number of normal basal sperms. Of the other two evaluated lines the one where bigger results of basal anomalous sperms obtained was the Lewis. On the other hand in the three evaluated lines were obtained a genotoxic ambient in the germinal cells with the use of the CF, being obtained bigger results of induction of anomalous sperms in the line of Lewis rats.

The fact that SD rats differed in a significant way in the variables of damage measured by the four assay with the other two evaluated lines, it reports a great utility, since the SD rats constitutes a heterogeneous line, with very similar response to that of the human populations, justifying its use in most of the pharmacological and toxicological preclinical studies (Arencibia & Rosario, 2009, 2010e; Arencibia *et al.*, 2010f).

Once selected the best mouse and rat as biomodels in genotoxicity assay we performed a comparison among both murine species among the endogenous rates and induced with cyclophosphamide keeping in mind the cytogenetic technical of the alkaline comet assay of peripheral blood leukocytes, micronucleus and chromosomic aberrations in bone marrow cells assay and lastly the sperm head morphology assay starting from samples of epididymis sperm.

The comet assay (Table 1) it showed that the best biomodel in both sexes was the SD rats differing significantly with the results obtained in Balb/c mice keeping in mind the spontaneous and induced values of damage to the DNA (Arencibia *et al.*, 2011e). The SD rats constitutes a heterogeneous line, reason why it will mimic with more degree of trust the possible effects that can achieve a drug evaluated by means of this assay in humans. Not being this way the mice of the Balb/c line, this mice line is isogenic and it experiences less genetic variability (Arencibia *et al.*, 2011e).

**Table 1:** Comparison between Balb/c mice and Sprague Dawley rats of both sexes subjected to the comet assay, according to induction of DNA damage of peripheral blood leukocytes.

Groups	Sex	Arbitrary Units	Level 0	Level 1	Level 2	Level 3	Level 4
			(Nucleoids %)				
**Balb/c mice of both sexes**							
Negative Control	F	49.51±10.24	56.00±8.01	39.29±7.49	4.01±4.17	0.60±0.68	0.10±0.05
	M	56.37±7.51	51.03±3.14	42.72±5.68	5.10±2.77	1.15±1.05	0.00±0.00
Vehicle Substance 1	F	57.23±10.20	50.82±9.32	42.13±2.22	6.05±5.28	1.00±0.93	0.00±0.00
	M	54.25±8.90	55.50±9.01	36.15±3.58	7.00±4.66	1.30±1.45	0.05±0.03
Vehicle Substance 2	F	52.15±10.31	57.47±3.76	34.27±8.58	6.90±4.50	1.36±0.46	0.00±0.00
	M	56.33±7.62	49.97±10.03	44.92±2.02	4.00±5.76	1.03±0.49	0.08±0.02
Positive Control (CF)‡	F	118.02±13.28*	23.33±5.22*	53.02±10.64*	10.41±4.99*	8.78±3.90*	4.46±1.43*
	M	112.69±14.11*	25.80±3.41*	52.78±11.98*	9.37±1.90*	7.03±2.01*	5.02±0.91*
**Sprague Dawley rats of both sexes**							
Negative Control	F	34.80±10.24a	79.14±4.32a	11.86±5.02a	5.37±3.10a	2.32±1.01a	1.31±1.00a
	M	33.46±7.51a	80.10±5.22a	11.23±5.56a	5.00±2.89	2.45±1.20	1.22±0.98a
Vehicle Substance 1	F	32.56±10.20a	80.32±7.83a	11.11±4.28a	5.25±2.99a	2.33±1.73a	0.99±0.34a
	M	35.03±8.90a	78.21±9.10a	13.19±4.77a	4.98±2.33a	2.60±1.26	1.02±0.83a
Vehicle Substance 2	F	33.19±10.31a	80.46±6.59a	10.78±5.11a	5.17±3.15a	2.29±1.51	1.30±0.99a
	M	33.44±7.62a	79.75±3.83a	12.19±4.99a	4.96±2.51	2.07±1.65	1.28±1.01a
Positive Control (CF)‡	F	101.45±13.28*a	35.56±3.55*a	43.47±3.44*a	10.31±3.91*	6.08±2.80*a	4.78±2.46*
	M	106.83±14.11*a	32.76±4.88*a	44.67±4.77*a	10.56±3.68*	7.00±2.00*	5.01±2.51*

CF (cyclophosphamide)

‡ Administration by i.p. route

* *p<*0.05 (Comparison against the negative control in the same species, Mann Whitney U test).

a=*p<*0.05 (It differs when comparing among species keeping in mind the same variable in the same experimental group, Mann Whitney U test). (X mean; S.D. standard deviation, for the two analyzed series).

In the micronucleus assay (Table 2) the ideal biomodel was the Balb/c mouse, keeping in mind the lowest values in spontaneous micronucleus and the induced higher values, being more susceptible than the SD rats to the CF (Arencibia *et al.*, 2011f). Demonstrating that the Balb/c mice by means of the mechanism of endogenous formation of micronucleus in erythrocytes of bone marrow cells are more stable genetically that the SD rats (Arencibia *et al.*, 2011g), being a strong predominant factor in this study again the fact that mouse line is isogenic, obtaining low rates of genetic variations and epigenetics among individuals (Arencibia *et al.*, 2011f).

**Table 2:** Comparison of the basal and induced frequency of micronucleus in erythrocytes of bone marrow between Balb/c mice and SD rats of both sexes.

Group	n	PE/NE^d^	MN-PE (%)^d^	MN^d^
**Balb/c mice**				
Male				
Negative Control	10	1.18±0.01**a**	0.16±0.03**a**	23**a**
Vehicle Substance 1	10	1.16±0.04**a**	0.18±0.04**a**	26**a**
Vehicle Substance 2	10	1.19±0.05	0.18±0.04**a**	25**a**
Positive Control (CF)‡	10	0.87±0.03* **a**	1.82±0.89* **a**	258* **a**
Female				
Negative Control	10	1.15±0.05**a**	0.13±0.04**a**	19**a**
Vehicle Substance 1	10	1.17±0.02**a**	0.14±0.07**a**	20**a**
Vehicle Substance 2	10	1.19±0.04	0.17±0.08	24
Positive Control (CF)‡	10	0.85±0.02* **a**	1.65±0.77* **a**	233* **a**
**Sprague Dawley rats**				
Male				
Negative Control	10	1.20±0.03	0.19±0.05	27
Vehicle Substance 1	10	1.19±0.04	0.21±0.02	30
Vehicle Substance 2	10	1.18±0.06	0.15±0.06	22
Positive Control (CF)^‡^	10	0.90±0.03*	1.68±0.92*	241*
Female				
Negative Control	10	1.19±0.06	0.22±0.01	32
Vehicle Substance 1	10	1.21±0.02	0.20±0.02	29
Vehicle Substance 2	10	1.18±0.04	0.17±0.05	25
Positive Control (CF)^‡^	10	0.89±0.04*	1.74±1.03*	250*

CF (cyclophosphamide).

‡ Administration by i.p. route.

d 2000 cells/animal analysed

* *p<*0.05 comparison with the control, ANOVA Test, X mean; S.D standard deviation, for the two analysed series. (DDetermination in 2 000 PE/animal, *p<*0.01 (comparison with the control, Chi Squared χ^2^ no parametric test, for the two analyzed series).

a=*p<*0.05 (It differs when comparing among species keeping in mind the same variable in the same experimental group, using the same statistic test).

On the other hand in the chromosomic aberrations assay (Table 3) the best experimental biomodel as for the spontaneous indexes was the Balb/c mice, experienced less cells with aberrations, but the rats SD demonstrated to be more susceptible to the CF, inducing bigger number of structural chromosomic aberrations (Arencibia *et al.*, 2011f). Then SD rats could be used in this assay to determine genotoxic activity of new drugs that induce damage to the DNA by alquilant effect, as chemical with clastogenic effects (Hayashi *et al.*, 1994; Higashikuni & Sutou, 1995), allowing the determination of the mechanism of damage of new drugs.

**Table 3:** Results of the comparison of the spontaneous and induced frequency of chromosomic aberrations in bone marrow between Balb/c mice and SD rats of both sexes.

Groups	MI (%)^a^	Cells with poliploidy^b^	Gaps^b^	Number of cells with aberrations^b^
**Balb/c mice**				
Male				
Negative Control	5.65±0.56^a^	1	4	7^a^
cyclophosphamide (50 mg/kg, i.p)	3.89±0.24* a	14** a	47** a	175** a
Vehicle Substance 1	5.49±0.53^a^	0	6	8^a^
Vehicle Substance 2	5.86±0.20^a^	0^a^	7^a^	10^a^
Female				
Negative Control	5.98±0.22^a^	1	6	8a
cyclophosphamide (50 mg/kg, i.p)	3.93±0.84* a	18** a	44** a	192** a
Vehicle Substance 1	4.98±0.79	0^a^	5	8^a^
Vehicle Substance 2	5.12±0.63	1	2^a^	9^a^
**Sprague Dawley rats**				
Male				
Negative Control	4.93±0.09	2	6	17
cyclophosphamide (50 mg/kg, i.p)	3.58±0.43*	23**	62**	220**
Vehicle Substance 1	5.12±0.18	2	7	18
Vehicle Substance 2	5.29±0.25	3	4	23
Female				
Negative Control	4.81±0.10	1	5	18
cyclophosphamide (50 mg/kg, i.p)	3.40±0.26*	28**	69**	246**
Vehicle Substance 1	4.97±0.21	3	8	19
Vehicle Substance 2	5.19±0.32	1	6	20

a X ± S.D., 10 000 total cells/group/serie for a total of 20 000 evaluated cells

* *p<*0.05; ANOVA Test.

** *p<*0.01

b Chi Squared χ^2^ non parametric test. Comparison against negative control for both tests in the same species.

a=*p<*0.05 (It differs when comparing among species keeping in mind the same variable in the same experimental group, using the same statistic test).

When comparing the basal and induced results in the sperm head morphology assay (Table 4) among both species it was obtained that the line of Balb/c mice differs with the SD rats in all the analysed variables (Arencibia *et al.*, 2011k). Experiencing this line of mice the lower spontaneous values as for the analysis of the genotoxic variables, but in turn this line experiment bigger spermatic concentration that the SD rats (Arencibia *et al.*, 2011k). When analysing the results of the induced variables it is observed that equally in the case of the morphology of the sperm head the Balb/c mice is more susceptible to the CF (Arencibia *et al.*, 2011i), but when measuring the cytotoxicity by means of the spermatic concentration they turned out to be more resistant to the damage induced by the CF than the SD rats (Arencibia *et al.*, 2011k). In this study we obtained as a final result that the best experimental biomodel to be the Balb/c mice (Arencibia *et al.*, 2011i), differing significantly with the results obtained in the SD rats, keeping in mind the spontaneous and induced values in the spermatic concentration and the frequency of morphological anomalous sperms in epididymis (Arencibia *et al.*, 2011k).

**Table 4:** Comparison between Balb/c mice and SD rats in the sperm head morphology assay and spermatic concentration in epididymis.

Group	n	Normal	Abnormal	Amorphous	Bananas	Without Hook	Two Tails	Concentration* (10^6^) Cells/mL
**Balb/c mice**								
NC	20	489.3±5.8^a^	10.7±5.8^a^	6.2±5.7^a^	1.2±1.7^a^	2.0±3.8^a^	0.3±1.5^a^	2.27±0.2^a^
VS1	20	483.2±8.3^a^	16.8±5.9^a^	12.0±1.6^a^	1.7±0.3^a^	2.6±3.1^a^	0.5±0.9^a^	2.24±0.2^a^
VS2	20	487.6±3.7^a^	12.4±3.7^a^	7.1±5.2^a^	1.7±1.2^a^	3.1±2.7^a^	0.5±0.2^a^	2.25±0.3^a^
CF ‡	20	399.5±12.3* ^a^	100.5±6.3^*a^	39.1±6.8* ^a^	22.1±3.7* ^a^	29.9±6.1^*^	9.4±2.0* ^a^	0.84±0.5*^a^
**Sprague Dawley rats**								
NC	20	460.4±19.4	39.6±10.4	14.3±4.3	10.4±8.2	13.7±6.8	1.2±0.7	2.18±0.5
VS1	20	460.6±8.3	39.4±8.3	16.6±6.6	9.3±4.4	11.8±3.7	1.7±0.9	2.09±0.3
VS2	20	455.3±17.5	44.7±13.5	18.4±3.8	11.4±7.9	12.6±6.1	2.3±1.7	1.97±0.2
CF ‡	20	408.0±7.0*	92.0±7.0*	27.6±8.0*	30.0±2.9*	30.5±7.0*	3.9±0.5*	0.81±0.3*

(NC: Negative Control, VS1: Vehicle Substance 1, VS2: Vehicle Substance 2, CF: Cyclophosphamide).

‡ Administration by i.p. route, during five consecutive days. Determinations in 500 cells/animal.

* *p<*0.05 (comparison with the negative control in the same species, ANOVA Test).

a=*p<*0.05 (It differs when comparing among species keeping in mind the same variable in the same experimental group, ANOVA Test). (X mean; S.D. standard deviation, for the two analysed series).

The opposing differences as for the expression of the genotoxic damage caused by the CF could be since in response to different levels of expression of the genes that code for the cytochrome P-4501A1 enzyme in the liver, the mice and rats differ genetic and epigenetically when keeping in mind this fundamental hepatic enzyme in the I phase of the xenobiotic metabolism that participates in the metabolism of the CF in the liver when being used as cytostatic drug (Amri *et al.*, 1986; Bell *et al.*, 1993; Jana *et al.*, 1998).

For this drug to be metabolized, it is necessary the activation in the hepatic microsomal, in a first step to hydroxide-cyclophosphamide, transforming spontaneously to aldo-phosphamide, and later on in the target cells it becomes mustard phosphor amide, of which four metabolites arises: -mustard phosphor amide (activate), achroleine, carboxyl-phosphamide and 4-cecophosphamide that are barely active. The CF is inactivated for microsomal and hepatic enzymes with active participation of the P-450 cytochrome (Arencibia *et al.*, 2010f).

For the first time it was performed a deep characterization, endorsed statistically by several biomodels keeping in mind their efficiency (Arencibia *et al.*, 2011, 2011h, 2011j). Besides not existing until the moment a harmonization as for the best biomodel able to detect substances with low or little genotoxic effect, that which when suggesting the use of a species and line in particular will allow to these assays a bigger sensibility and robustness (Arencibia *et al.*, 2011, 2011h, 2011j). In this study the characterization from the point of view of the endogenous damage to the DNA for several genotoxicity mechanisms, contributed to knowledge of the biomodels that markets our country, being of importance to support in equal forms the export toward other countries interested in evaluating its products using our biomodels.
